# Lower Diet Quality Associated with Subclinical Gastrointestinal Inflammation in Healthy United States Adults

**DOI:** 10.1016/j.tjnut.2024.02.030

**Published:** 2024-03-01

**Authors:** Yasmine Y Bouzid, Stephanie MG Wilson, Zeynep Alkan, Charles B Stephensen, Danielle G Lemay

**Affiliations:** 1 Department of Nutrition, University of California, Davis, Davis, CA; 2 USDA-ARS Western Human Nutrition Research Center, Davis, CA, United States; 3 Texas A&M AgriLife, Institute for Advancing Health Through Agriculture, College Station, TX, United States

**Keywords:** diet, inflammation, humans, inflammatory bowel disease, vegetables

## Abstract

**Background:**

Higher diet quality has been associated with lower risk of developing inflammatory bowel disease, but associations between diet and gastrointestinal (GI) inflammation in healthy adults prior to disease onset are understudied.

**Objectives:**

The purpose of this project was to examine associations between reported dietary intake and markers of GI inflammation in a healthy adult human cohort.

**Methods:**

In a cross-sectional observational trial of 358 healthy adults, participants completed ≤3 unannounced 24-h dietary recalls using the Automated Self-Administered Dietary Assessment Tool and a Block 2014 Food Frequency Questionnaire to assess recent and habitual intake, respectively. Those who provided a stool sample were included in this analysis. Inflammation markers from stool, including calprotectin, neopterin, and myeloperoxidase, were measured by ELISA along with LPS-binding protein from plasma.

**Results:**

Recent and habitual fiber intake was negatively correlated with fecal calprotectin concentrations (*n* = 295, *P* = 0.011, 0.009). Habitual soluble fiber intake was also negatively correlated with calprotectin (*P* = 0.01). Recent and habitual legume and vegetable intake was negatively correlated with calprotectin (*P* = 0.013, 0.026, 0.01, 0.009). We observed an inverse correlation between recent Healthy Eating Index (HEI) scores and calprotectin concentrations (*n* = 295, *P* = 0.026). Dietary Inflammatory Index scores were calculated and positively correlated with neopterin for recent intake (*n* = 289, *P* = 0.015). When participants with clinically elevated calprotectin were excluded, recent and habitual fiber, legume, vegetable, and fruit intake were negatively correlated with calprotectin (*n* = 253, *P* = 0.00001, 0.0002, 0.045, 0.001, 0.009, 0.001, 0.004, 0.014). Recent total HEI score was inversely correlated with subclinical calprotectin (*P* = 0.003).

**Conclusions:**

Higher diet quality may be protective against GI inflammation even in healthy adults.

This trial was registered at clinicaltrials.gov as NCT02367287.

## Introduction

The incidence of inflammatory bowel disease (IBD) and colorectal cancer is increasing in industrialized countries where diets are marked by increased consumption of low quality foods rich in saturated fat and deplete in fruits and vegetables [1]. As chronic gastrointestinal (GI) inflammation underlies the development of IBD and colorectal cancer [2,3], associations between diet and subclinical GI inflammation may be relevant to risk of these chronic diseases. Whereas higher diet quality, including high-fiber content from fruits and vegetables, is associated with reduced risk of developing IBD [4,5], diets abundant in fast food with high saturated fat content are associated with development of both Crohn’s disease (CD) and ulcerative colitis (UC) [6]. Therefore, it seems likely that diet influences regulators of GI inflammation prior to the onset of disease, yet these associations are underinvestigated in healthy adults.

Diets rich in refined grains and animal fats are generally low in fiber, which can lead to increased gut permeability and loss of regulatory, anti-inflammatory immune functions [7]. In murine models, low fiber diets disrupt GI immune homeostasis through loss of bacterial diversity in the microbiota, increased mucus-degrading bacteria, and decreased epithelial integrity [8]. Conversely, high-fiber diets have been consistently associated with increased abundance of microbes from the genus *Bifidobacterium* [9,10]. Fiber-fermenting *Bifidobacterium* species produce short-chain fatty acids (SCFAs) that interact with T-regulatory cells to control inflammation, and their extinction increases risk of colitis [11]. Thus, if low quality diets reduce the abundance of microbes that regulate the ability of T-regulatory cells to control inflammation, we expect to find elevated markers of inflammation in individuals consuming low quality diets.

Other mechanisms by which diet may influence GI inflammation include direct modulation of inflammatory pathways by saturated fatty acids and metabolic support of intestinal epithelial cells by SCFAs. Saturated fatty acids induce inflammation via toll-like receptor pathways that have been primed by LPS [12]. Diet and microbially derived metabolites, such as SCFAs, may provide metabolic support of intestinal epithelial cells, promoting metabolic flexibility [2]. Impaired metabolic flexibility may contribute to impaired barrier function and impaired intestinal regeneration, contributing to increased susceptibility to inflammatory stimuli [2,13]. Adults without active inflammatory conditions provide an ideal population to study how certain dietary components may drive or protect from these early inflammatory processes.

We selected reliable molecular markers of GI inflammation to measure and analyze associations with diet. Calprotectin is a highly sensitive marker of GI inflammation produced by neutrophils migrating to compromised sites in the gut epithelium [14] that is used clinically to measure inflammation in patients with active inflammatory disease. Other markers of GI inflammation include myeloperoxidase, which is also released by neutrophils and catalyzes the production of reactive oxygen species in the inflamed gut [15]. Neopterin is a robust fecal marker of GI inflammation produced by human macrophages when stimulated by the proinflammatory cytokine interferon-γ [16,17]. Increased gut permeability allows LPS from Gram-negative bacteria to translocate to the bloodstream and increase concentrations of LPS-binding protein (LBP) in plasma [18]. Elevation of these markers is consistent with GI inflammation and higher gut permeability.

The relationship between diet and GI inflammation in healthy adults is understudied as most research has been conducted on adults with active clinical inflammatory conditions rather than healthy individuals prior to disease onset. Thus, we examined associations between diet quality and markers of GI inflammation in a healthy cohort of nearly 350 United States adults. In the study design article for this cohort, Primary Hypothesis #2 predicted that higher Healthy Eating Index scores, and other measures of diet quality, would be associated with lower gut inflammation [19]. In addition to testing that hypothesis, we also hypothesized that fiber, fruit, and vegetable intake would be negatively associated with GI inflammation marker concentrations (calprotectin, myeloperoxidase, and neopterin) and with gut permeability marked by LBP. Saturated fat intake was expected to be positively associated with markers of inflammation and gut permeability. We also examined the association between the Dietary Inflammatory Index score and these markers of GI health.

## Methods

### Study design and recruitment

**FIGURE 1:**
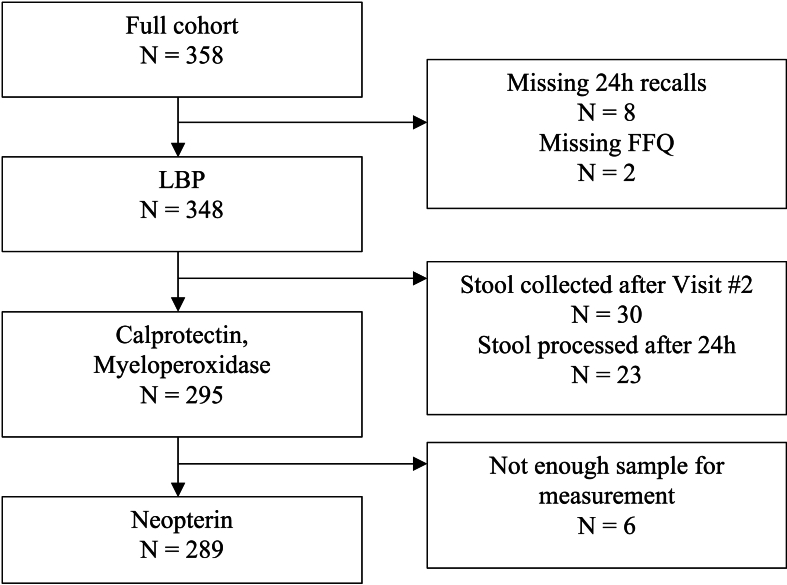
CONSORT of distributions included in this analysis based on completion of dietary data and availability of plasma and stool samples.

Study participants were recruited for the USDA Nutritional Phenotyping Study (clinicaltrials.gov identifier: NCT02367287) conducted at the USDA Western Human Nutrition Research Center in Davis, CA from May 2015 to July 2019 [19]. The study was approved by the University of California, Davis Institutional Review Board. Healthy adults between the ages of 18 and 65 y who had no diagnoses of chronic disease were recruited and stratified by sex and BMI ranging from 18.5 to 45 kg/m^2^. Potential participants were excluded if they had been previously diagnosed with GI disease, had a history of GI surgery, or had taken antibiotics in the previous month. Fasting blood samples and stool samples were collected from participants at a test day visit 10 to 14 d after an initial intake visit [20]. Participants were included in the current analysis if they completed ≥2 at-home recalls that passed dietary quality control and/or completed the food frequency questionnaire (FFQ) and provided a fasting blood sample and/or stool sample (see CONSORT diagram in Figure 1).

### Dietary data collection

Dietary data collection and cleaning has been previously described [19]. Briefly, participants completed 24-h recall training with study personnel using the National Cancer Institute (NCI) Automated Self-Administered Dietary Assessment Tool (ASA24) [21]. Then, participants were prompted to complete 3 24-h recalls at home for 2 weekdays and 1 weekend day between their study visits (10–14-d period). A dietitian supervised data cleaning on open-ended text entries for 24-h recalls [22]. Block 2014 FFQs were completed under supervision of trained personnel at visit 2 to assess habitual intake over the previous year. Total fiber is reported in the 24-h recalls as grams consumed per day (averaged across available recalls). To normalize for caloric intake, we calculated fiber consumed per 1000 calories of intake. The FFQ reports both insoluble and soluble fiber, and soluble fiber per 1000 calories was selected for hypothesis testing because microbiota ferment this type more [23]. Vegetable (excluding and including legumes), legume, and fruit intake are reported in the 24-h recalls and FFQ using the Food Patterns Equivalents Database (FPED) [24]. FPED represents consumption as cup equivalents to evaluate alignment with Dietary Guidelines for Americans recommendations. To normalize for caloric intake, we calculated cup equivalents consumed per 1000 calories of intake. Saturated fat, reported in grams consumed per day by 24-h recalls and FFQ, was normalized for caloric intake as grams per 1000 calories of intake.

### Stool sample collection

Participants provided stool samples as previously described [25]. Participants transported samples to the research center on cold packs as soon as possible for processing, including homogenization, and storage at −80°C.

### Plasma sample collection

Plasma was processed from fasting blood collected in heparin tubes immediately after the blood draw. Aliquots were stored at −80°C.

### Quantification of markers of GI inflammation and gut permeability

As described previously, fecal calprotectin, fecal myeloperoxidase, fecal neopterin and plasma LBP were measured using ELISA kits [26].

### Calculation of dietary indices

Healthy Eating Index (HEI) 2015 scores for 24-h recalls were calculated using macros provided by NCI [27]. HEI-2015 scores were provided for the FFQ by NutritionQuest who sources the Block FFQ. FFQ Total Dietary Inflammatory Index (DII) scores were also provided by NutritionQuest and calculated based on the population-based DII developed by Shivappa et al. [28]. DII scores were also calculated for the 24-h recalls using the R package DietaryIndex [29].

### Statistical analysis

R was used for statistical analysis and visualizations. Linear regression was used to examine associations between dietary components and stool calprotectin, myeloperoxidase, neopterin, and plasma LBP. Distribution transformations were described previously [26]. Briefly, fecal calprotectin was transformed using an ln(x + 3) transformation, fecal myeloperoxidase and plasma LBP were transformed using an ln(x + 1) transformation, and fecal neopterin was transformed using a Box Cox transformation. One-way analysis of variance was used to assess differences in mean concentrations of GI inflammation markers between 2 sexes (male and female), 3 age groups (18–33.99, 34–49.99, and 50–65 y), and 3 BMI groups (<25, 25–29.9, and 30–39.9 kg/m^2^). Sex was included as a categorical factor and age and BMI were included as numerical factors as covariates in regression models when examining associations between dietary intake variables and markers of GI inflammation. The false discovery rate was controlled by adjusting *P* values for multiple hypothesis testing using the method of Benjamini and Hochberg [30]. The statistical analyses reported in this paper are provided in a GitHub repository at https://github.com/YasmineYBouzid/diet_guthealth_publication.

## Results

### Participant characteristics

As previously reported, there were 164 male and 184 female participants with GI inflammation data [22,26]. Mean BMI for the 348 participants included in this study was 27.28 ± 4.9 kg/m^2^ with a range from 18.04 to 43.87 kg/m^2^. Mean age was 40.51 ± 13.7 y with a range from 18 to 66 y. The number of participants included in the distributions for each marker of GI inflammation and reasons for exclusion are shown in Figure 1.

### Distribution of markers in healthy adults and relationship with age, sex, and BMI

**FIGURE 2:**
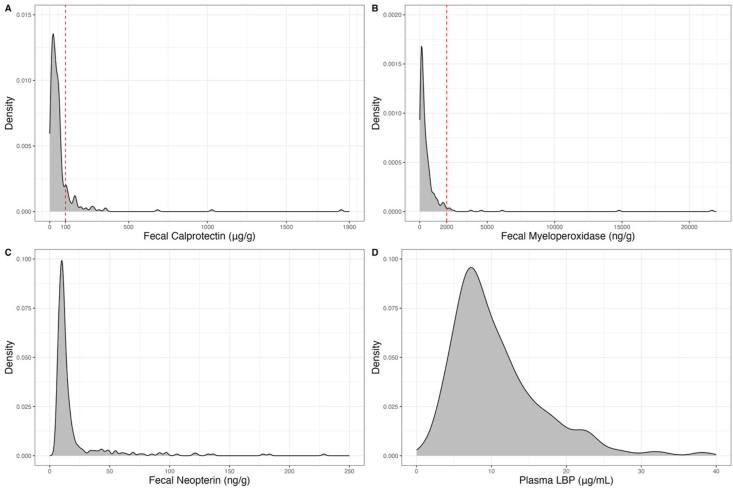
Density distributions of GI inflammation markers (A) calprotectin, (B) myeloperoxidase, (C) neopterin, and (D) LBP. Red dotted lines indicate threshold for clinical inflammation for calprotectin (100 μg/g) and myeloperoxidase (2000 ng/g). GI, gastrointestinal.

Untransformed distributions of fecal calprotectin, fecal myeloperoxidase, fecal neopterin, and plasma LBP are shown in Figure 2. Even among healthy participants, measurements of these markers have a broad range. Using thresholds (>100 μg/g calprotectin; >2000 ng/g myeloperoxidase) suggested by the assay manufacturer, some of the healthy participants exhibited frank GI inflammation. Calprotectin clinical thresholds for fresh stool are usually 50 μg/g, but we selected the higher threshold of 100 μg/g because the samples were frozen and thawed, which can cause cell lysis and falsely elevated calprotectin concentrations. Clinical thresholds have not been established for neopterin and plasma LBP.

After appropriate transformation of these outcome variables, we examined the relationship between participant characteristics used to stratify recruitment (age, sex, and BMI) and GI inflammation marker concentrations. There were no significant differences between males and females for calprotectin and myeloperoxidase; however, females had higher mean neopterin and LBP concentrations (Supplemental Figure S1). There were no significant differences in GI inflammation markers between age groups (Supplemental Figure S2). Only LBP concentrations differed between BMI categories: those with ≥30 kg/m^2^ had significantly higher LBP than both those between 25.0 and 29.9 kg/m^2^ and those with <25 kg/m^2^ (Supplemental Figure S3).

### Dietary fiber and GI health

We first addressed our hypothesis that fiber intake would be negatively associated with GI inflammation and intestinal permeability. Calprotectin concentrations were negatively correlated with recent and habitual total fiber intake as well as habitual soluble fiber intake (Table 1). Habitual total fiber intake was negatively correlated with myeloperoxidase but not after adjustment for multiple hypothesis testing. Recent total fiber intake was negatively correlated with fecal neopterin but not after adjustment for multiple hypothesis testing.

**TABLE 1:** Associations between fiber intake and GI inflammation and permeability markers adjusted for age, sex, and BMI

	Data source	Adjusted *R*^2^	Slope	*P*	Adjusted *P*
Calprotectin (*n* = 295)					
Total fiber (g/1000 kcal)	ASA24	0.025	−0.036	0.004 ∗∗	0.011 ∗
Total fiber (g/1000 kcal)	FFQ	0.032	−0.03	0.001 ∗∗	0.009 ∗∗
Soluble fiber (g/1000 kcal)	FFQ	0.025	−0.09	0.004 ∗∗	0.01 ∗
Myeloperoxidase (*n* = 295)					
Total fiber (g/1000 kcal)	ASA24	−0.007	−0.013	0.379	0.607
Total fiber (g/1000 kcal)	FFQ	0.009	−0.023	0.02 ∗	0.073
Soluble fiber (g/1000 kcal)	FFQ	0.0006	−0.065	0.082	0.148
Neopterin (*n* = 289)					
Total fiber (g/1000 kcal)	ASA24	0.047	−0.032	0.016 ∗	0.065
Total fiber (g/1000 kcal)	FFQ	0.028	−0.004	0.678	0.872
Soluble fiber (g/1000 kcal)	FFQ	0.017	0.003	0.918	0.918
LPS-binding protein (*n* = 348)					
Total fiber (g/1000 kcal)	ASA24	0.219	0.007	0.193	0.807
Total fiber (g/1000 kcal)	FFQ	0.216	0.003	0.525	0.983
Soluble fiber (g/1000 kcal)	FFQ	0.217	0.013	0.335	0.983

Significant relationships between outcomes and dietary components highlighted in bold (α = 0.05). Adjusted *P* values from multiple hypothesis testing correction using Benjamini-Hochberg method are also shown. ∗ *P* < 0.05, ∗∗ *P* < 0.01, ∗∗∗ *P* < 0.001.

Abbreviations: ASA24, Automated Self-Administered 24-hour Dietary Assessment Tool; BMI, body mass index; FFQ, food frequency questionnaire; GI, gastrointestinal.

### Dietary vegetable, fruit, and saturated fat and GI health

We next tested whether intake of vegetables, fruit, and/or saturated fat was associated with GI health. Recent and habitual intake of vegetables, whether excluding or including legumes, was negatively correlated with fecal calprotectin concentrations (Table 2). Recent and habitual legume intake was negatively correlated with calprotectin concentrations. Habitual fruit intake was also negatively correlated with calprotectin concentrations. Habitual vegetable intake, excluding legumes, recent legume, habitual total vegetable, and habitual fruit intake were negatively correlated with fecal myeloperoxidase but not after adjustment for multiple hypothesis testing. Recent vegetable intake, excluding legumes, was also negatively correlated with fecal neopterin but not after adjustment for multiple hypothesis testing. Saturated fat intake was not significantly associated with markers of GI inflammation or intestinal permeability.

**TABLE 2:** Associations between vegetable, fruit, and saturated intake and GI inflammation and permeability markers adjusted for age, sex, and BMI.

	Data source	Adjusted *R*^2^	Slope	*P*	Adjusted *P*
Calprotectin (*n* = 295)					
Vegetables, excluding legumes (cup eq./1000 kcal)	ASA24	0.025	−0.254	0.004 ∗∗	0.011 ∗
	FFQ	0.022	−0.2	0.007 ∗∗	0.013 ∗
Legumes (cup eq./1000 kcal)	ASA24	0.017	−1.05	0.016 ∗	0.026 ∗
	FFQ	0.029	−1.09	0.002 ∗∗	0.009 ∗∗
Total vegetables (cup eq./1000 kcal)	ASA24	0.033	−0.276	0.001 ∗∗	0.01 ∗
	FFQ	0.027	−0.02	0.003 ∗∗	0.009 ∗∗
Total fruit (cup eq./1000 kcal)	ASA24	0.0004	−0.096	0.323	0.369
	FFQ	0.014	−0.248	0.027 ∗	0.04 ∗
Saturated fat (g/1000 kcal)	ASA24	-0.002	0.007	0.646	0.646
	FFQ	0.0009	−0.01	0.288	0.288
Myeloperoxidase (*n* = 295)					
Vegetables, excluding legumes (cup eq./1000 kcal)	ASA24	-0.005	−0.118	0.261	0.607
	FFQ	0.008	0.197	0.024 ∗	0.073
Legumes (cup eq./1000 kcal)	ASA24	0.004	−1.02	0.048 ∗	0.382
	FFQ	-0.0007	−0.689	0.104	0.156
Total vegetables (cup eq./1000 kcal)	ASA24	-0.002	−0.149	0.141	0.566
	FFQ	0.008	−0.181	0.021 ∗	0.073
Total fruit (cup eq./1000 kcal)	ASA24	-0.009	−0.038	0.741	0.911
	FFQ	0.006	−0.277	0.035 ∗	0.079
Saturated fat (g/1000 kcal)	ASA24	-0.01	−0.005	0.797	0.911
	FFQ	-0.003	−0.015	0.151	0.194
Neopterin (*n* = 289)					
Vegetables, excluding legumes (cup eq./1000 kcal)	ASA24	0.041	−0.184	0.048 ∗	0.088
	FFQ	0.035	−0.113	0.148	0.444
Legumes (cup eq./1000 kcal)	ASA24	0.028	−0.05	0.914	0.914
	FFQ	0.038	0.661	0.08	0.358
Total vegetables (cup eq./1000 kcal)	ASA24	0.04	−0.172	0.055	0.088
	FFQ	0.031	−0.068	0.333	0.5
Total fruit (cup eq./1000 kcal)	ASA24	0.037	−0.167	0.101	0.135
	FFQ	0.031	−0.12	0.304	0.5
Saturated fat (g/1000 kcal)	ASA24	0.029	0.008	0.615	0.703
	FFQ	0.028	−0.001	0.9	0.918
LPS-binding protein (*n* = 348)					
Vegetables, excluding legumes (cup eq./1000 kcal)	ASA24	0.215	0.013	0.751	0.98
	FFQ	0.215	−0.001	0.964	0.983
Legumes (cup eq./1000 kcal)	ASA24	0.217	−0.166	0.377	0.807
	FFQ	0.216	0.099	0.517	0.983
Total vegetables (cup eq. /1000 kcal)	ASA24	0.215	0.005	0.894	0.98
	FFQ	0.215	0.002	0.936	0.983
Total fruit (cup eq. /1000 kcal)	ASA24	0.217	0.034	0.404	0.807
	FFQ	0.216	0.02	0.656	0.983
Saturated fat (g/1000 kcal)	ASA24	0.216	−0.004	0.521	0.833
	FFQ	0.215	0.0006	0.873	0.983

Significant relationships between outcomes and dietary components highlighted in bold (α = 0.05). Adjusted *P* values from multiple hypothesis testing correction using Benjamini-Hochberg method are also shown. ∗ *P* < 0.05, ∗∗ *P* < 0.01, ∗∗∗ *P* < 0.001.

Abbreviations: ASA24, Automated Self-Administered 24-hour Dietary Assessment Tool; BMI, body mass index; FFQ, food frequency questionnaire; GI, gastrointestinal.

### Dietary indices and GI inflammation

**FIGURE 3:**
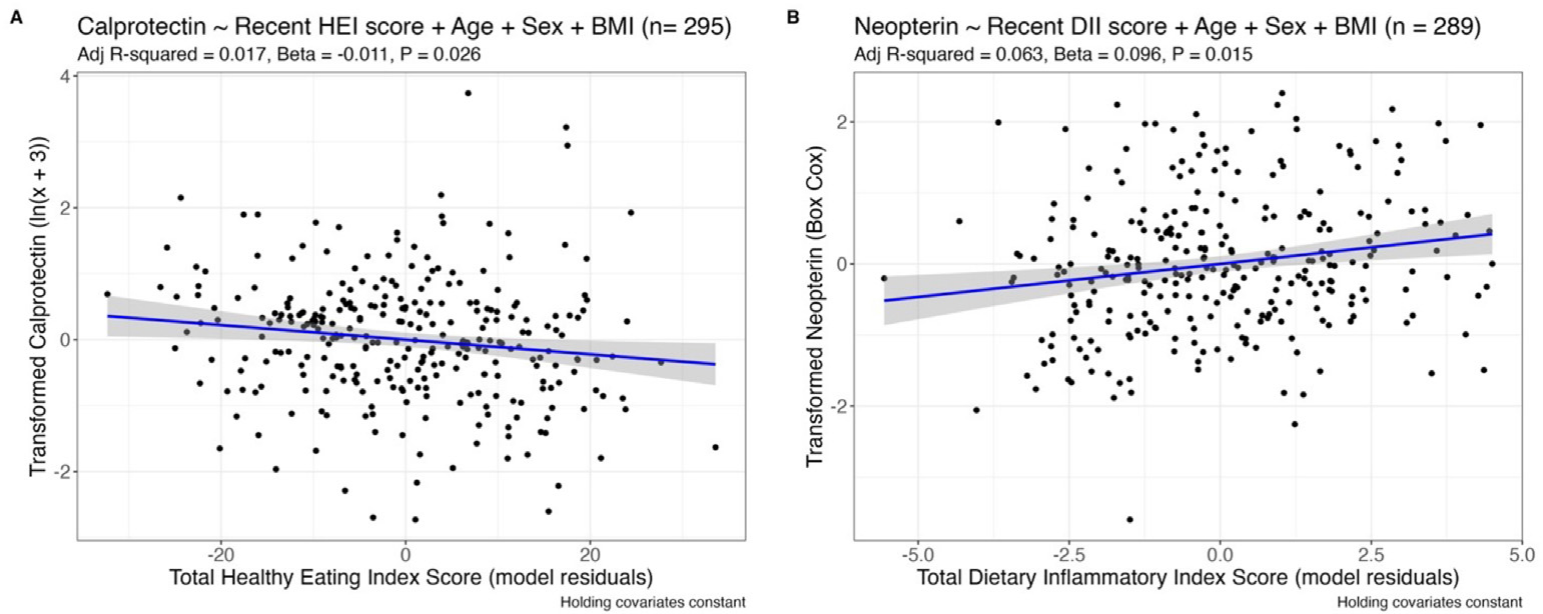
Partial regressions of (A) transformed fecal calprotectin and (B) transformed neopterin with significant predictors. (A) The y-axis is the residuals of transformed fecal calprotectin given covariates—sex, age, BMI–(holding them constant) and x-axis is the residuals of total Healthy Eating Index score (holding covariates constant). (B) The y-axis is the residuals of transformed fecal neopterin given covariates—sex, age, BMI–(holding them constant) and x-axis is the residuals of total Dietary Inflammatory Index score (holding covariates constant). Blue line shows best fit and gray shading shows standard error (95% confidence interval). BMI, body mass index; DII, Dietary Inflammatory Index; HEI, Healthy Eating Index.

Dietary indices summarize whole diets into a single numeric variable. We tested the association of the total HEI score and the total DII score with measures of GI inflammation and permeability in these healthy adults. Total HEI scores can range from 0 to 100, with 100 indicating full compliance with USDA Dietary Guidelines. The DII score can range from −8.87 to +7.98, with positive and negative scores respectively indicating pro- and anti-inflammatory potential. Total HEI scores from recent intake were negatively correlated with calprotectin concentrations (Table 3 and Figure 3). As expected, recent DII scores were positively correlated with fecal neopterin concentrations (Table 3 and Figure 3). Recent HEI and habitual DII scores were negatively and positively correlated, respectively, with neopterin concentrations but not after adjustment for multiple hypothesis testing.

**TABLE 3:** Associations between dietary indices and GI markers, adjusted for age, sex, and BMI

	Data source	Adjusted *R*^2^	Slope	*P*	Adjusted *P*
Calprotectin (*n* = 295)					
Total Healthy Eating Index score	ASA24	0.017	−0.011	0.016 ∗	0.026 ∗
	FFQ	0.001	−0.007	0.271	0.288
Total Dietary Inflammatory Index score	ASA24	0.006	0.047	0.099	0.131
	FFQ	0.003	0.026	0.182	0.234
Myeloperoxidase (*n* = 295)					
Total Healthy Eating Index score	ASA24	−0.007	−0.005	0.355	0.607
	FFQ	−0.009	−0.003	0.642	0.642
Total Dietary Inflammatory Index score	ASA24	−0.01	−0.002	0.957	0.957
	FFQ	−0.007	0.02	0.374	0.421
Neopterin (*n* = 289)					
Total Healthy Eating Index score	ASA24	0.041	−0.01	0.049 ∗	0.088
	FFQ	0.031	−0.007	0.313	0.5
Total Dietary Inflammatory Index score	ASA24	0.063	0.096	0.002 ∗∗	0.015 ∗
	FFQ	0.042	0.041	0.04 ∗	0.357
LPS-binding protein (*n* = 348)					
Total Healthy Eating Index score	ASA24	0.215	0.00005	0.98	0.98
	FFQ	0.215	−0.00006	0.983	0.983
Total Dietary Inflammatory Index score	ASA24	0.217	0.01	0.4	0.807
	FFQ	0.215	0.002	0.802	0.983

Significant relationships between outcomes and dietary scores highlighted in bold (α = 0.05). Adjusted *P* values from multiple hypothesis testing correction using Benjamini-Hochberg method are also shown. ∗ *P* < 0.05, ∗∗ *P* < 0.01, ∗∗∗ *P* < 0.001.

Abbreviations: ASA24, Automated Self-Administered 24-hour Dietary Assessment Tool; BMI, body mass index; FFQ, food frequency questionnaire; GI, gastrointestinal.

### Dietary intake and subclinical GI inflammation

**FIGURE 4:**
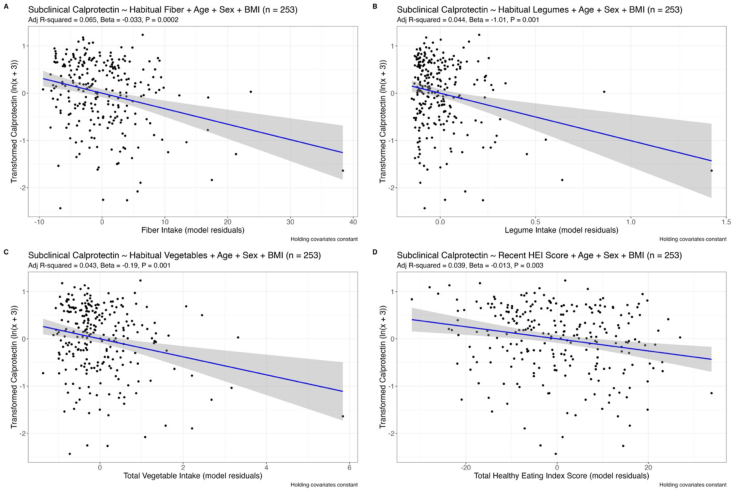
Partial regression plots of the residuals of transformed fecal “subclinical” calprotectin given covariates—sex, age, BMI (holding them constant)—and the residuals of reported dietary intake (holding covariates constant) where the reported dietary intake is (A) habitual fiber intake, (B) habitual legume intake, (C) habitual total vegetable intake, or (D) recent total HEI score. Only participants with subclinical fecal calprotectin (*n* = 253) are included in this analysis. Blue line shows best fit and gray shading shows standard error (95% confidence interval). BMI, body mass index; HEI, Healthy Eating Index.

Participants suffering from acute GI inflammation may not be responsive to diet. Therefore, we retested our specific hypotheses after excluding data from participants with GI markers above thresholds positive for clinical inflammation (>100 μg/g calprotectin; >2000 ng/g myeloperoxidase). Exclusion using these thresholds removed 14.2% (*n* = 42) of the participants from the calprotectin distribution and 3.1% (*n* = 9) from the myeloperoxidase distribution. Recent and habitual fiber, soluble fiber, legume, and vegetable intake were negatively correlated with subclinical calprotectin, as expected (Table 4). Recent and habitual fruit intake were negatively correlated with subclinical calprotectin. Total HEI scores calculated from the recent diet, but not the habitual diet, were negatively correlated with subclinical calprotectin. We visualized the associations between habitual fiber, legume, and vegetable intake and recent HEI scores with subclinical calprotectin (Figure 4). Habitual fiber and soluble fiber, recent and habitual legume, habitual total vegetable, and habitual fruit intake were negatively correlated with subclinical myeloperoxidase but not after adjustment for multiple hypothesis testing. Total DII scores were not significantly associated with subclinical calprotectin or myeloperoxidase. The remaining dietary variables were not associated with either GI marker (Table 4). In summary, the dietary variables that were associated with fecal calprotectin in the full cohort (TABLE 1, TABLE 2, TABLE 3), were associated with subclinical fecal calprotectin along with recent fruit intake (Table 4).

**TABLE 4:** Assocations between dietary components and sublinical markers of GI inflammation (calprotectin and myeloperoxidase) adjusted for age, sex, and BMI

	Data Source	Adjusted R^2^	Slope	*P* value	Adjusted *P* value
Calprotectin (*n* = 253)					
Total fiber (g/1000 kcal)	ASA24	0.085	−0.05	0.000001 ∗∗∗	0.00001 ∗∗∗
	FFQ	0.065	−0.033	0.00002 ∗∗∗	0.0002 ∗∗∗
Soluble fiber (g/1000 kcal)	FFQ	0.054	−0.101	0.00009 ∗∗∗	0.0004 ∗∗∗
Vegetables, excluding legumes (cup eq./1000 kcal)	ASA24	0.02	−0.187	0.012 ∗	0.02 ∗
	FFQ	0.036	−0.199	0.001 ∗∗	0.002 ∗∗
Legumes (cup eq./1000 kcal)	ASA24	0.012	−0.763	0.034 ∗	0.045 ∗
	FFQ	0.044	−1.01	0.0004 ∗∗∗	0.001 ∗∗
Total vegetables (cup eq./1000 kcal)	ASA24	0.026	−0.203	0.005 ∗∗	0.009 ∗∗
	FFQ	0.043	−0.19	0.0004 ∗∗∗	0.001 ∗∗
Fruit (cup eq./1000 kcal)	ASA24	0.034	−0.254	0.002 ∗∗	0.004 ∗∗
	FFQ	0.022	−0.244	0.009 ∗∗	0.014 ∗
Saturated fat (g/1000 kcal)	ASA24	0.008	0.023	0.07	0.08
	FFQ	0.004	−0.011	0.119	0.153
Total Healthy Eating Index score	ASA24	0.039	−0.013	0.001 ∗∗	0.003 ∗∗
	FFQ	0.003	−0.008	0.138	0.156
Total Dietary Inflammatory Index score	ASA24	0.005	0.038	0.113	0.123
	FFQ	−0.000004	0.019	0.237	0.237
Myeloperoxidase (*n* = 286)					
Total fiber (g/1000 kcal)	ASA24	−0.0003	−0.024	0.077	0.268
	FFQ	0.009	−0.024	0.017 ∗	0.076
Soluble fiber (g/1000 kcal)	FFQ	0.003	−0.069	0.043 ∗	0.077
Vegetables, excluding legumes (cup eq./1000 kcal)	ASA24	−0.007	−0.108	0.258	0.344
	FFQ	0.001	−0.151	0.06	0.089
Legumes (cup eq./1000 kcal)	ASA24	0.004	−0.963	0.04 ∗	0.268
	FFQ	0.005	−0.836	0.031 ∗	0.077
Total vegetables (cup eq./1000 kcal)	ASA24	−0.003	−0.138	0.135	0.268
	FFQ	0.004	−0.148	0.038 ∗	0.077
Fruit (cup eq./1000 kcal)	ASA24	−0.003	−0.163	0.132	0.268
	FFQ	0.011	−0.03	0.011 ∗	0.077
Saturated fat (g/1000 kcal)	ASA24	−0.009	0.014	0.398	0.455
	FFQ	−0.008	−0.009	0.358	0.46
Total Healthy Eating Index score	ASA24	−0.005	−0.007	0.168	0.268
	FFQ	−0.009	−0.005	0.448	0.492
Total Dietary Inflammatory Index score	ASA24	−0.011	0.01	0.75	0.75
	FFQ	−0.01	0.014	0.492	0.492

Significant relationships between outcomes and dietary components highlighted in bold (α = 0.05). Adjusted *P* values from multiple hypothesis testing correction using Benjamini-Hochberg method are also shown. ∗ *P* < 0.05, ∗∗ *P* < 0.01, ∗∗∗ *P* < 0.001.

Abbreviations: ASA24, Automated Self-Administered 24-hour Dietary Assessment Tool; BMI, body mass index; FFQ, food frequency questionnaire; GI, gastrointestinal.

## Discussion

National nutrition and health surveys historically have not included stool collections, leading to a limited understanding of the relationship between diet and markers of GI inflammation in healthy populations. Thus, we sought to fill this important scientific gap by assessing the relationship between diet (recent, habitual) and several indicators of GI inflammation including fecal calprotectin, myeloperoxidase, and neopterin. We found that recent and habitual fiber, vegetable, and legume and habitual fruit intake were negatively correlated with fecal calprotectin, which was consistent with a systematic review that reported inverse associations between fiber, vegetable, and fruit intake and incidence of IBD [31]. Our evaluation of dietary indices showed that recent total HEI scores were negatively correlated with calprotectin, and recent DII scores were positively correlated with neopterin. When excluding participants above subclinical thresholds for calprotectin and myeloperoxidase, we observed inverse associations between recent and habitual fiber, legume, vegetable, and fruit intake and calprotectin. These findings support our hypotheses that components contributing to high diet quality would be inversely associated with GI inflammation markers whereas components contributing to low diet quality would be positively associated.

Overall diet quality, as measured by the HEI, was negatively associated with fecal calprotectin. The relationship of HEI and calprotectin was even stronger when participants with acute GI inflammation were excluded. Diet and microbiome may be somewhat decoupled during acute GI inflammation. In the healthy state, obligate anaerobes convert dietary carbon sources into beneficial metabolites like SCFAs. A dysbiotic microbiome arises when there is an increase in harmful microbes or a loss of these beneficial microbes and their healthy metabolites, causing epithelial damage and inflammation (reviewed in [1]). In these cases, microbes may be thriving on endogenously produced carbon sources, rather than dietary sources. For example, in macaques with a type of UC, fecal metatranscriptomes provided evidence of increased host production of mucins, increased consumption and cross-feeding of these fucosylated mucins, and increased adherence of a pathogen to the mucosa relative to healthy controls despite the fact that both healthy and sick animals were on the same diet [32]. This shift of microbial fermentation from dietary to host glycans may explain why diet–microbiome relationships were more significant when participants with acute GI inflammation were excluded from analysis.

Among healthy individuals who were not experiencing acute GI inflammation, we observed a spectrum of GI health measured by fecal markers, and this spectrum was found to be associated with overall diet quality. HEI scores were associated with calprotectin and had a strong negative correlation with subclinical calprotectin. This is consistent with findings from a Netherlands-based study comparing GI inflammation in patients with IBD and irritable bowel syndrome to healthy controls, where Dutch Healthy Eating Index 2015 scores from a 1-mo FFQ were higher in healthy controls compared with patients. A negative association was also detected between diet quality scores and calprotectin in patients with IBD [33]. Regarding inflammatory potential of the diet, a study in patients with CD and healthy controls in China found that patients with CD consumed diets with higher DII scores (greater proinflammatory potential) than healthy controls and that scores positively correlated with fecal calprotectin [34]. We did not observe associations between DII scores and fecal calprotectin in our healthy cohort, but we did find a positive correlation between DII scores and fecal neopterin. Our findings suggest inflammation markers may be disparately affected by diet and bioactivity of dietary components.

Regarding specific dietary components, recent and habitual intake of fiber, legumes, vegetables, and fruit was negatively associated with calprotectin in individuals with subclinical GI inflammation. Reduction of systemic markers of inflammation including C-reactive protein and plasma IL-6 has been associated with high-fiber consumption [35]. Consumption of fiber-rich foods may support intestinal epithelial health by increasing microbial fermentation products such as SCFAs, which act both as fuel to epithelial cells and signaling molecules to help prevent activation of inflammatory cascades [36]. Decreased fecal calprotectin was found in a 3-mo supplementation trial with obese adults who consumed 16 g of inulin fiber coupled with dietary guidance to consume inulin-rich vegetables [37]. Hence, we also examined associations between these food groups and GI inflammation markers to include other potentially beneficial factors in the analysis. Legumes contain bioactive compounds like hydrosylates and (poly)phenols that inhibit activation of the nuclear factor κβ pathway. In a mouse model, soybean peptides decreased concentrations of inflammatory cytokines in LPS-induced colitis [38]. Among patients with CD in clinical remission, daily leafy green vegetable intake was associated with lower fecal calprotectin concentrations [39]. Our findings are consistent with previous beneficial associations with anti-inflammatory properties of plant-based foods.

Interestingly, the DII was less predictive of GI inflammation than the HEI. The HEI relationship is unsurprising as it is in alignment with the general recommendation of improving dietary patterns to reduce systemic inflammation [40]. This recommendation may extend to the intestinal environment as well as a Mediterranean-like dietary pattern was negatively associated with fecal calprotectin [41]. The HEI evaluates dietary quality by analyzing the intake of food groups and specific nutrients in accordance with the Dietary Guidelines for Americans, whereas the DII assesses the inflammatory potential of a dietary pattern. The DII includes 45 food parameters, a percentile scoring system, and inclusion of multiple markers for inflammatory effect scores [28]. The DII notably differs from the HEI in that it includes specific nutrients and inflammation-modulating foods to determine the inflammatory potential of the diet. We note that the tool used to calculate DII from ASA24 did not include 17 of the 45 parameters, notably missing flavonoid subclasses and polyphenol-rich foods, and thus, may not accurately capture the anti-inflammatory potential of recent dietary intake. Additionally, inflammatory effect scores for the DII were developed from blood inflammatory markers, rather than intestinal markers, which may explain why HEI dietary quality was more predictive of GI inflammation. As an assessment of overall dietary patterns, HEI may better capture the anti-inflammatory benefits of nutrient synergies and nutritional “dark matter” [42].

Our findings suggest that GI inflammation may be influenced more by acute diet than long-term dietary patterns. The HEI from recent 24-h recalls, indicating recent diet quality, showed a negative association with GI inflammation, whereas the HEI from habitual dietary data obtained through the FFQ did not. This difference may be partly attributed to differences in how each tool estimates food consumption. Recalls allow for a detailed assessment of dietary intake by incorporating specific food items and portion weights compared with FFQs, which aggregate food items and use less specific food portion sizes. Improved measurement accuracy with 24-h recalls is likely as stronger correlations to nutritional biomarkers have been found with recent diet compared with habitual diet in the United Kingdom–based European Prospective Investigation into Cancer Norfolk Study [43]. Moreover, acute changes to diet are known to alter microbial composition, although shifts are temporary [44,45]. Community changes can alter production of microbially derived metabolites [46], which in turn may influence the luminal, mucosal, and epithelial environments [2]. However, these short-term dietary studies often include extreme diets or novel components [45]. As our study utilized a cross-sectional design, it is more likely that the relationship between GI inflammation and acute dietary intake is from improved measurement of food consumption, which could be tested in future studies with validated nutritional biomarkers.

Contrary to our hypothesis, we did not observe a positive association between saturated fat intake and markers of inflammation and gut permeability. Our hypothesis was based on the proinflammatory effect of saturated fatty acids both systemically [47] and in the intestine [48]. Our null finding with saturated fat suggests that intestinal epithelial cells in healthy adults have an adequate capacity to metabolize saturated fatty acids, which keeps intracellular concentrations low and prevents the generation of proinflammatory mediators [2,49,50]. As we observed reduced calprotectin with increased fiber, vegetable, and fruit intake, this cellular metabolic flexibility could be supported in part by anti-inflammatory microbial derivatives from increased consumption of fiber-rich foods. With reduced fiber-rich food consumption, we suspect that acute and persistent saturated fatty acid exposure may have a more pronounced proinflammatory effect.

High plasma LBP concentrations, a biomarker of elevated LPS and GI permeability, were detected with higher BMI and in females. LBP was not associated with diet quality or intake of fiber, saturated fat, or other dietary variables after adjustment for BMI and sex. LPS is implicated in obesity development as demonstrated by work in murine models and humans. Administration of LPS in CD14-knockout mice on a high-fat diet did not lead to the development of obesity [51], underscoring the importance of LPS-induced inflammation to weight gain. In humans, overweight and obese adults undergoing weight management programs showed improved gut permeability [52,53] and reduced LPS-producing bacteria [53]. Although the entry of LPS into systemic circulation is facilitated by the joint transport of triglycerides [54], it is interesting that saturated fat intake in our study was not associated with LBP. Importance of dietary fat in LPS-mediated metabolic dysregulation was previously shown in several mouse studies, where mice dosed with LPS only demonstrated increased LPS when on a high-fat diet compared with a normal unpurified diet [51,55]. As we did not detect an association with dietary factors, we propose that factors related to lipid metabolism, such as HDL, which serves as a potent LPS scavenger, may have had a stronger impact on plasma LBP [56].

In this study, although the directionality of GI markers was either supportive of hypotheses or nonsignificant, the GI markers were not consistent with each other in association with diet. This may be partly due to different stability of the fecal markers. All 3 fecal biomarkers have been demonstrated to be stable for multiple days in stool samples [17,57,58], and we did exclude the 23 samples that had >24 h between collection and storage at −80°C. However, although fecal calprotectin and neopterin have some stability even at room temperature, myeloperoxidase has not been demonstrated to have such stability, and this may be why myeloperoxidase was not significant in any analysis.

Another reason for differences among biomarkers is that they indicate activation of different types of immunity. If type I immunity is triggered (viral infection), it is expected there would be a higher macrophage and lower neutrophil response (potentially more neopterin, less calprotectin/myeloperoxidase). If type 3 immunity is triggered (extracellular bacteria), it is expected there would be a higher neutrophil and lower macrophage response (potentially more calprotectin/myeloperoxidase, less neopterin). All immune responses involve a mix of immune cells, and both (type I and type 3) are active in the GI tract to prevent infection from the microbiota. The trend for associations between diet and fecal neopterin in this cohort could be indicative of a weak correlation between diet and encounters with GI viruses; however, these results were not significant after multiple hypothesis testing correction. The more robust association of fecal calprotectin with fiber, legume, vegetable, and fruit intake and total dietary quality suggests that diet may potentially have a greater impact on type 3 immunity (extracellular bacteria), which is consistent with the fact that diet can directly influence the gut microbiome. That LBP was not associated with diet further suggests that, in healthy people, the neutrophil infiltration in response to extracellular bacteria is sufficient to maintain gut barrier function.

This study has several limitations. First, causal relationships between diet and inflammation markers cannot be made as this study utilized a cross-sectional design. We cannot distinguish between acute and chronic inflammation because we did not collect multiple stool samples; participants above the clinical thresholds exhibited frank inflammation at a single time point. Second, dietary intake was self-reported, which introduces measurement error including systemic recall bias. Lastly, GI inflammation can be induced by a range of environmental stimuli. Although we controlled for age, sex, and BMI in our statistical models, there may be additional biological factors affecting GI inflammation for which we did not account.

In summary, we found that higher diet quality was associated with lower concentrations of GI inflammation markers. GI inflammation markers are not typically measured in healthy populations; thus, this study provides valuable information about the distribution of multiple GI inflammation markers in adults without clinical inflammatory conditions. As our study utilized multiple 24-h recalls and an FFQ, we were able to differentiate possible acute effects from habitual effects of diet on inflammatory outcomes. This is noteworthy as many health outcomes are assessed in relation to FFQs. As diet can both alleviate and exacerbate inflammatory pathways in the intestine, it is essential to better understand the influence of recent and habitual diet before the onset of chronic conditions. Future interventions may seek to test whether specific food groups or improvements to overall dietary quality differentially improve GI inflammation, with further consideration of how dietary effects may differ across the clinical spectrum.

## Acknowledgments

We thank Eduardo Cervantes, Ellen Bonnel, and the USDA Nutritional Phenotyping Study Team for assisting in data collection, the USDA Bioanalytical Support Lab for sample management and stool processing, and Sarah Spearman for assistance with fecal sample analysis.

## Author contributions

The authors’ responsibilities were as follows – DGL, CBS: designed research; YYB, ZA: conducted research; YYB: analyzed data; YYB, SMGW, DGL: wrote the paper; ZA, CBS: edited the paper; DGL: had primary responsibility for final content; and all authors: read and approved the final manuscript.

### Conflict of interest

CBS reports financial support was provided by USDA-ARS Western Human Nutrition Research Center. DGL reports funding from the California Prune Board and the California Dairy Research Foundation and board membership and travel reimbursement from the International Milk Genomics Consortium. CBS is on the editorial board of the *Journal of Nutrition*. All other authors report no conflicts of interest.

## Funding

This research was supported by USDA Agricultural Research Service grant 2032-51530-026-00D. The project described was supported by the National Center for Advancing Translational Sciences (NCATS), National Institutes of Health (NIH), through grant UL1 TR001860.

## Data availability

Requests for data from the USDA ARS WHNRC Nutritional Phenotyping Study used in this analysis should be made via an e-mail to the senior WHNRC author on the publication of interest. Requests will be reviewed quarterly by a committee consisting of the study investigators. All scripts are available at https://github.com/YasmineYBouzid/diet_guthealth_publication.
