# Supplementary material for: Lower Diet Quality Associated with Subclinical Gastrointestinal Inflammation in Healthy United States Adults
Source: J Nutr. 2024 Mar 1;154(4):1449–60. doi: 10.1016/j.tjnut.2024.02.030 (PMC11347802; doi:10.1016/j.tjnut.2024.02.030)
Supplement: Multimedia component 1 [file mmc1.docx]

Supplementary Materials for

**Lower Diet Quality Associated with Subclinical Gastrointestinal Inflammation in Healthy U.S. Adults**

Yasmine Y. Bouzid ^1^, Stephanie M.G.Wilson ^2,3^, Zeynep Alkan ^2^, Charles B. Stephensen ^1,2^, and Danielle G. Lemay ^1,2^ *

^1^ Department of Nutrition, University of California, Davis, Davis, CA 95616

^2^ USDA-ARS Western Human Nutrition Research Center, Davis, CA 95616

^3^ Texas A&M AgriLife, Institute for Advancing Health Through Agriculture, College Station, TX 77845

***** Correspondence: Danielle G. Lemay; danielle.lemay@usda.gov

Contents:

Supplementary Figures 1-3


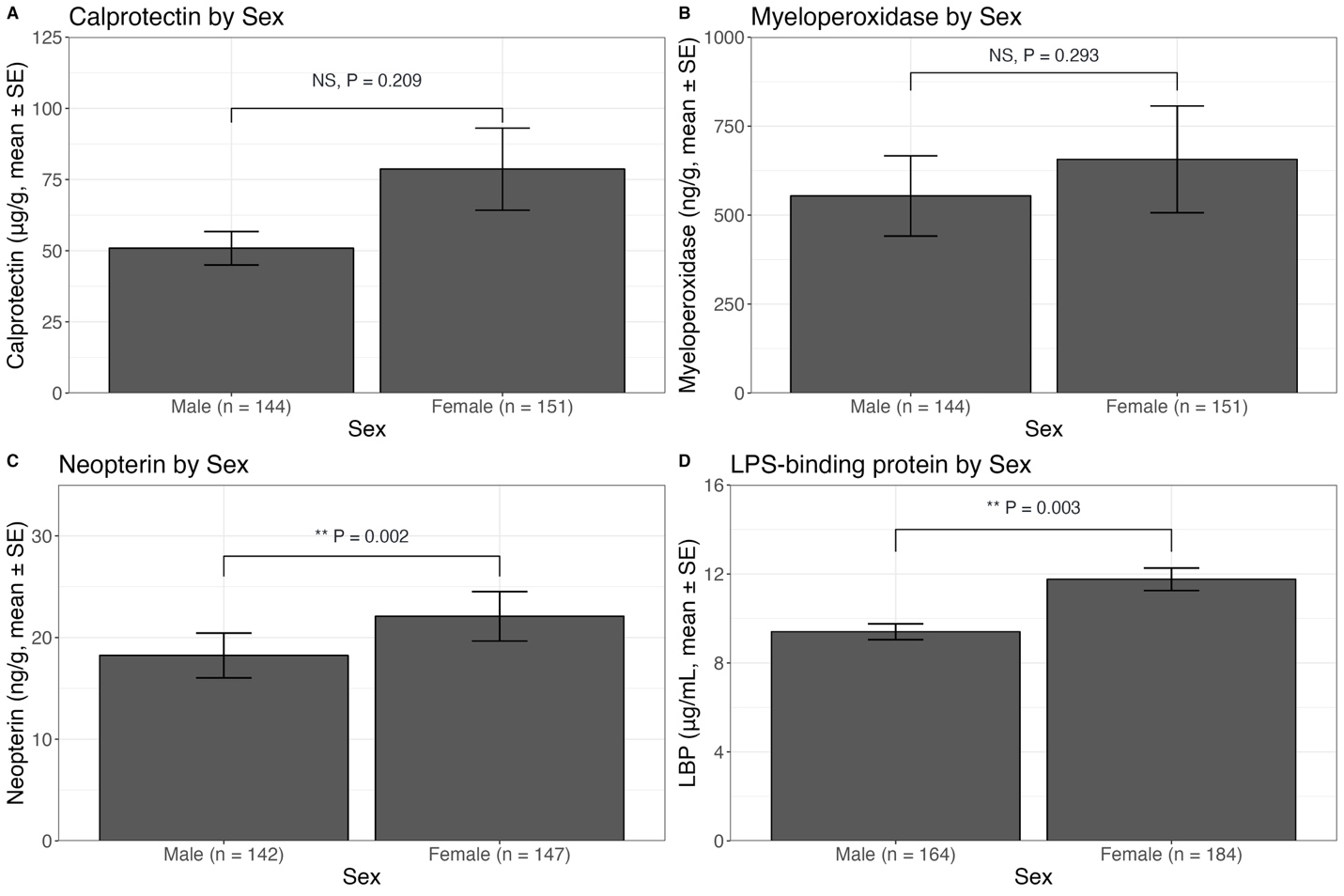


*Figure S1*. Differences in GI inflammation (A) calprotectin (B) myeloperoxidase (C) neopterin and gut permeability markers (D) LBP, between males and females by ANOVA (α = 0.05; NS = not significant, * P < 0.05, ** P < 0.01, *** P < 0.001)

*
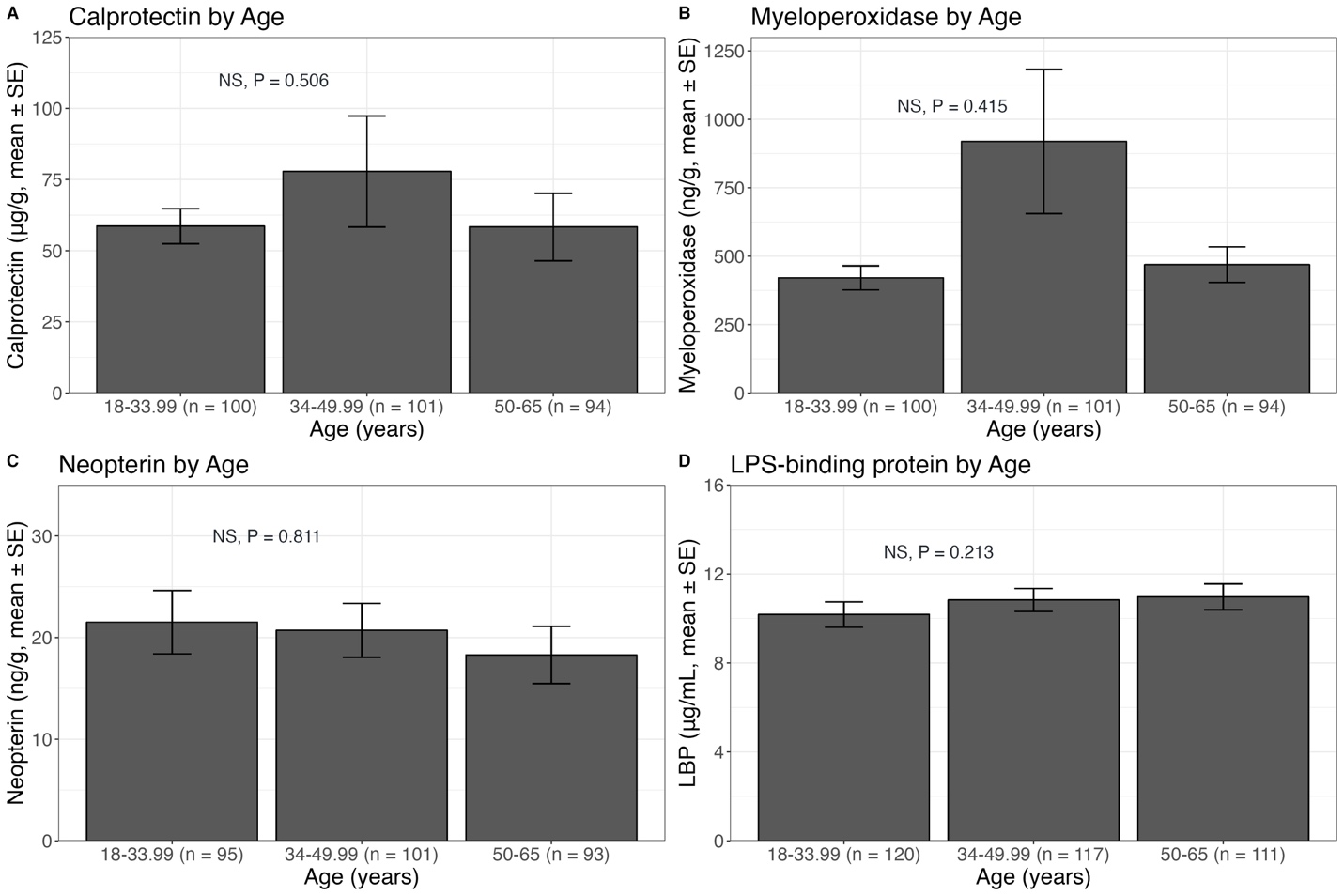
*

*Figure S2*. Differences in GI inflammation (A) calprotectin (B) myeloperoxidase (C) neopterin and gut permeability markers (D) LBP, between age groups by ANOVA (α = 0.05; NS = not significant, * P < 0.05, ** P < 0.01, *** P < 0.001)

*
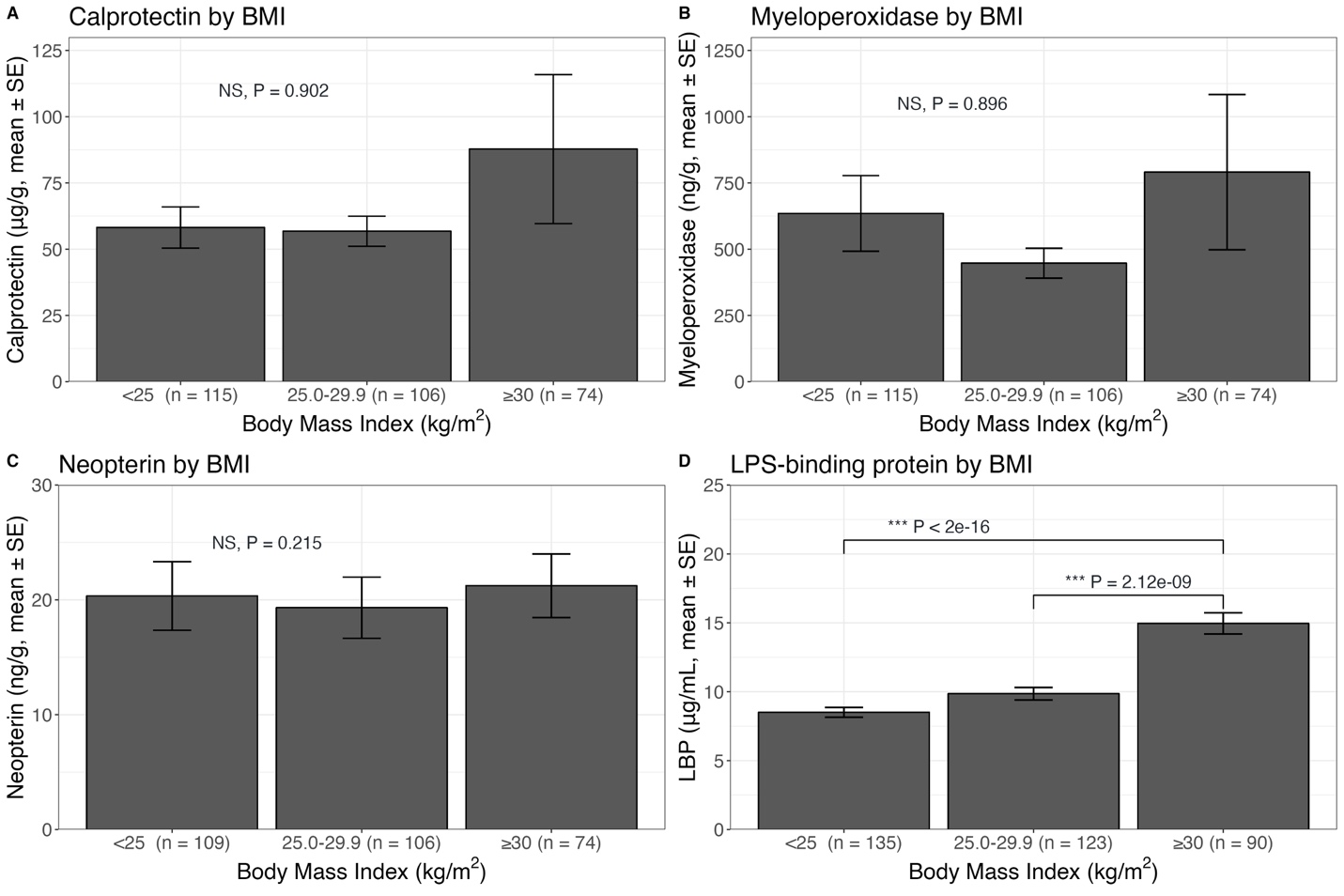
*

*Figure S3*. Differences in GI inflammation (A) calprotectin (B) myeloperoxidase (C) neopterin and gut permeability markers (D) LBP, between BMI category by ANOVA (α = 0.05; NS = not significant, * P < 0.05, ** P < 0.01, *** P < 0.001)
